# Correction: Assessment of psychological terror and its impact on mental health and quality of life in medical residents at a reference medical center in Mexico: A cross-sectional study

**DOI:** 10.1371/journal.pone.0315207

**Published:** 2024-12-04

**Authors:** Víctor Manuel Enriquez Estrada, Neftali Eduardo Antonio-Villa, Omar Yaxhemen Bello-Chavolla, Carlos Fredy Cuevas-García, Pedro Luis Vargas Gutiérrez, Irma Sau-Yen Corlay Noriega, Luis Rey García-Cortés

There are errors in the author affiliations. The correct affiliations are as follows:

Víctor Manuel Enriquez Estrada^1,2^, Neftali Eduardo Antonio-Villa^3^, Omar Yaxhemen Bello-Chavolla^4^, Carlos Fredy Cuevas-García^1^, Pedro Luis Vargas Gutiérrez^5^, Irma Sau-Yen Corlay Noriega^1^, Luis Rey García-Cortés^6^

**1** UMAE, Hospital de Especialidades Centro Médico Nacional Siglo SXXI, Instituto Mexicano del Seguro Social, Ciudad de México, México, **2** Hospital General de Zona 197, Instituto Mexicano del Seguro Social, Estado de México, México, **3** Departamento de Endocrinología, Instituto Nacional de Cardiología Ignacio Chávez, Ciudad de México, México, **4** Dirección de Investigación, Instituto Nacional de Geriatría, Ciudad de México, México, **5** Coordinación De Planeación y Enlace Institucional, Instituto Mexicano del Seguro Social, Estado de México, Oriente, México, **6** Coordinación Auxiliar Médica de Investigación en Salud, Instituto Mexicano del Seguro Social, Estado de México, Oriente, México
