# Supplementary material for: Assessment of psychological terror and its impact on mental health and quality of life in medical residents at a reference medical center in Mexico: A cross-sectional study
Source: PLoS One. 2023 Dec 6;18(12):e0295138. doi: 10.1371/journal.pone.0295138 (PMC10699592; doi:10.1371/journal.pone.0295138)
Supplement: S1 File — (DOCX) [file pone.0295138.s002.docx]

**Highlights**

**Assessment of psychological terror and its impact on mental health and quality of life in medical residents at a reference medical center in Mexico: A cross-sectional study**

- We estimated the prevalence of psychological terror (the highest degree of workplace harassment) and its independently associated factors relating to anxiety, depression, and quality of life in a sample of medical residents enrolled in a reference medical center in Mexico City.
- Nearly one in every five medical residents evaluated in our study displayed features compatible with psychological terror.
- We observed that surgical specialties and residents in their second or fifth year had a higher propensity to experience psychological terror, a pattern which was more evident in women.
- Perpetrators of mobbing were most commonly medical residents at higher hierarchical positions, with medical staff coming up in second place.
- Increased anxiety levels and decreased quality of life in mental health among medical residents were the main correlates associated with psychological terror.
